# Model for Teaching the Helping Babies Survive Course Series and Advanced Life Support in Obstetrics Workshops to Rural Skilled Birth Attendants in Uganda

**DOI:** 10.4314/ahs.v21i4.44

**Published:** 2021-12

**Authors:** Christina Kinnevey, David Douglas, Ann Larsen, Molly Wilson, Sarah Sams, Michael Kawooya, Fred Kirumira, Robert Douglas

**Affiliations:** 1 Touro University California; 2 Stanford University; 3 Sutter Davis Hospital; 4 OhioHealth, Family Medicine Residency Program; 5 Makerere University, Radiology; Ernest Cook Ultrasound Research and Education Institute; 6 Ernest Cook Ultrasound Research and Education Institute; 7 Rad Impact

**Keywords:** Neonatal, Perinatal Care, Maternal Morbidity

## Abstract

**Background:** Uganda, like much of Sub-Saharan Africa and other underserved regions continues to face the challenge of high neonatal and maternal mortality. The Helping Babies Survive (HBS) course and the Advanced Life Support in Obstetrics (ALSO) provide hands on education to train providers in key life-saving interventions. A uterine balloon tamponade (UBT) procedure can be life-saving in the event of uterine bleeding. The purpose of this implementation research is to gain more insight into the effectiveness of a tailored down 5-day combined HBS-ALSO-UBT course. In this study, we found that a tailored down 5-day combined HBS-ALSO-UBT could be performed with significantly improved self-assessment in diagnosing and managing a wide range of peripartum conditions.

## Introduction

Uganda, like much of sub-Saharan Africa and other underserved regions continues to face the challenge of high maternal mortality. Despite some significant advancements in the last 20 years, in 2016, the maternal mortality rate was 336 per 100,000 live births.^1^ The goal of the 2016 Uganda Health Sector Development Plan (HSDP) is to reduce maternal mortality ratio to less than 219 per 100,000 live births by the year 2020.^1^ The United Nations Sustainable Development Goal (SDG) target by 2030 is less than 70 maternal deaths per 100,000 live births.2 Unfortunately, the rate of decline in Uganda maternal mortality is significantly slower than the global rate of decline. Forty-two percent of Ugandan maternal deaths are due to uncontrolled postpartum hemorrhage, which is the leading cause of maternal death worldwide.^1^,^3^

Similar to maternal mortality, significant reductions in neonatal mortality rate (NMR) will have to be made if Uganda is to meet the HSDP and SDG NMR goals. In 2017, the NMR was 27 per 1000 live births, far from the HSDP goal of <15 per 1000 live births by 2020 and the SDG goal of less than 12 per 1000 births by 2030.^1^,^2^ The majority of neonatal deaths occur due to birth complications and prematurity.^1^

Significant strides have been made in increasing the number of births in Uganda that are attended by a skilled birth attendant, the majority of whom being nurse midwives who received formal education and training to care for both mothers and babies in the peripartum period.^4^

However, one of the specific challenges identified by the Uganda Ministry of Health is that changes in clinical protocols for delivery of care not matching updates in training of health professionals.^1^,^5^ The Service Delivery Indicators for Uganda 2013 found that the majority of surveyed providers who were given vignettes on common maternal and neonatal complications only followed 1 of 5 of the correct clinical actions in management.^6^ Given that over 30% of neonatal deaths occur due to birth asphyxia, there is a strong need for improved neonatal resuscitation efforts, which have been shown to improve neonatal outcomes, especially when started within the “golden minute” immediately after delivery.7 A 2016 USAID report showed that while 84% of skilled birth attendants knew ventilation was required within the the golden minute, only 40% actually accomplished it and only 16% called for help and developed an emergency plan.^7^

The United States and other developed countries have long recognized simulated team training as a necessary requirement in learning how to approach emergency situations. The Neonatal Resuscitation Program (NRP) curriculum was developed in 1987 and the Advanced Life Support in Obstetrics (ALSO) curriculum in 1991 with the use of mannequins, mnemonics and hands on simulations. As challenges were repeatedly met adopting the NRP curriculum in low resource areas without neonatal intensive care units, a new curriculum was designed in 2010 that was specifically targeted for low resource settings, titled Helping Babies Breathe (HBB).^8^ Since 2010 when the curriculum was released, the course has been conducted in more than 80 countries, training over 500,000 providers.^9^ Additional courses were added to the HBB curriculum to include training in how to care for a baby after birth before discharge (Essential Care for Every Baby - ECEB) and specific training on how to care for preterm babies (Essential Care for Small Babies - ECSB). The three HBB, ECEB and ECSB courses are housed under one umbrella curriculum name titled Helping Babies Survive (HBS). The ALSO course, which includes a series of 5 lectures and 6 hands on workshops covering common birth complications has been taught in more than 60 countries worldwide since its rollout. However, given that surgical options for controlling postpartum hemorrhage refractory to uterotonic medications are limited in low resource rural areas, alternative methods were sought for these areas. The Mass General Global Health Innovation Laboratory developed a simple Uterine Balloon Tamponade (UBT) device kit consisting of a condom tied to a Foley catheter, which is inflated with clean water through a syringe and one-way valve. Increasing data from the past 7 years in multiple African countries supports the use of this ultra-low cost (<$5 U.S) UBT kit as a safe and effective option in management of severe postpartumhemorrhage.^3^

While the literature includes numerous studies that highlight efficacy of the HBS curriculum globally, there have not been as many published studies reviewing the ALSO course, particularly in combination with other neonatal curriculum. The overall purpose of this implementation research is to gain more insight into the effectiveness of a combined HBS-ALSO-UBT course. The purpose of this article is to 1. Propose a method for teaching the full HBS curriculum, ALSO workshops and training in use of UBT to rural Ugandan midwives in an efficient time frame and 2. Assess the self-efficacy of the course participants results of surveys of learners self-assessment of knowledge and confidence.

## Methods

### Procedures

This study was approved by the Uganda National Council for Science and Technology (UNCST). In 2015, a partnership was established between the Ernest Cook Ultrasound Research and Education Institute (ECUREI) in Uganda and Rad Impact, a United States based non-profit organization dedicated to improving maternal and child health abroad. ECUREI, in collaboration with the Uganda Protestant Medical Bureau (UPMB), selected 12 rural Ugandan prenatal clinics that had low antenatal care attendance rates. In January 2017 each of the 12 clinics plus an additional 7 clinics sent skilled birth attendants, primarily midwives, to attend ALSO, UBT, HBB and ECEB training courses in the capital city of Kampala. A U.S. team of 5 doctors, a nurse and a logistical support engineer traveled to Uganda to teach the courses in January 2017. In June 2018, a team of 4 doctors and a nurse traveled to Uganda and taught a different set of 17 midwives the same curriculum, except the ECSB course was added in place of some of the ALSO lectures and the duration of training each day was increased slightly. A summary of the courses taught each year are included in Table 1. Each year, pre and post course surveys were completed by the learners on self-assessment of confidence and knowledge. The 2017 surveys used a 3 point Likert scale to assess comfort level in diagnosis and management of common maternal and neonatal complications. In 2018, the ALSO/UBT pre and post survey was changed to a 6 point likert agreement scale to assess self efficacy in diagnosis and management of the peripartum conditions. The midwives had a heterogenous education background with prior education ranging from a few months to multiple years in duration. In addition, all surveys included general comments section.

**Table 1:** Educational activities performed in Session #1 (in 2017) and Session #2 (in 2018). Advanced Life Support in Obstetrics (ALSO); Uterine Balloon Tamponade (UBT); Helping Babies Breathe (HBB); Essential Care for Every Baby (ECEB); and, Essential Care for Small Babies (ECSB)

Educational Activities in Session #1	Educational Activities in Session #2
ALSO (workshops + lectures) UBT HBB ECEB	ALSO (workshops only) UBT HBB ECEB ECSB

### Statistical Analysis

The pre-course and post-course survey was completed by each SBA. Each outcome of interest was an ordinal variable. For example, in the 2017 survey, we assigned the number 1 to denote “not at all comfortable”, 2 to denote “somewhat comfortable”, or 3 to denote “very comfortable”. Given the small sample size and the use of ordinal data, we chose the nonparametric Wilxocon signed rank test. The null hypothesis is that the median of the differences between the pre-course self-assessment and post-course self-assessment is equal to zero. The alternative hypothesis is that the median of the differences between the pre-course self-assessment and the post-course self-assessment is not equal to zero. Each assessment is considered a separate hypothesis; therefore, a p-value of 0.05 is used to denote statistical significance. All statistical tests were performed with STATA version 14.1, College Station, TX.

## Results

Both in 2017 and 2018, the entire curriculum taught was covered in the span of 4.5 days, starting on a Monday and finishing on a Friday afternoon, allowing time for a graduation ceremony on Friday afternoon. Accounting for time for travel, midwives were away from their home clinics for 6–7 days, depending on the distance of their clinic from Kampala. U.S. physicians were gone for a total of 10 days including travel time, but only missed a total of 6 work weekdays and the time gone included 1.5 days for team tourism at the end of the trip.

**Figure 1:**
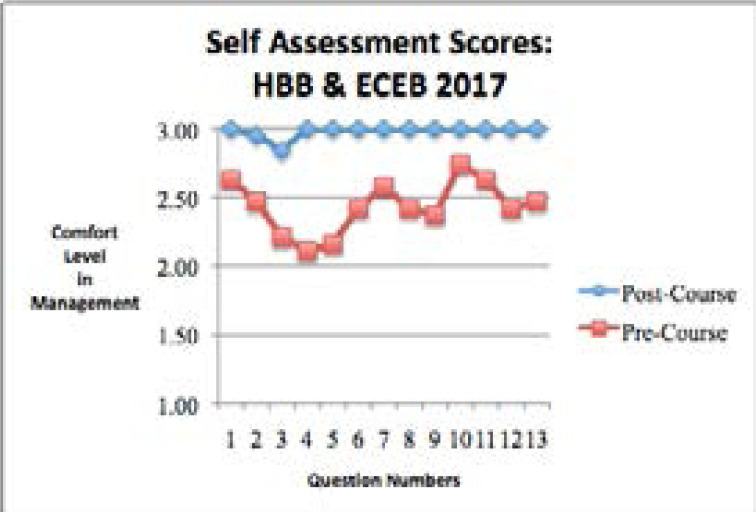
Self-assessment questions in the pre-training time period and the post-training time period for the 2017 Helping Babies Breathe (HBB) and Essential Care for Every Baby (ECEB) classes.

The 2017 self-assessment survey results from the HBB and ECEB course showed that the midwives' comfort level in diagnosis and management of neonatal conditions was significantly higher in the post-course assessment compared to the pre-course assessment for all 13 self-assessment questions (Table S1 and Figure 1).

**Table S1:** Self-assessment questions in the pre-training time period and the post-training time period for the 2017 Helping Babies Breathe (HBB) and Essential Care for Every Baby (ECEB) classes

Self-Assessment	Pre-training assessment (n=19) Mean ± SD	Post-training assessment (n=19) Mean ± SD	p-value
#1 Drying and stimulating a baby at birth	2.63 ± 0.50	3.00 ± 0.00	0.008*
#2 Using a checklist to prepare for birth	2.47 ± 0.61	2.95 ± 0.23	0.005*
#3 How to clean the bag and mask and suction after use	2.21 ± 0.79	2.84 ± 0.50	0.015*
#4 Management of baby that is not crying after birth	2.11 ± 0.66	3.00 ± 0.00	<0.001*
#5 How to ventilate a baby with a bag and mask	2.16 ± 0.60	3.00 ± 0.00	<0.001*
#6 How to provide eye care for a baby after birth	2.42 ± 0.61	3.00 ± 0.00	0.002*
#7 How to provide cord care for a baby after birth	2.58 ± 0.61	3.00 ± 0.00	0.008*
#8 How to protect babies from serious bleeding after birth	2.42 ± 0.61	3.00 ± 0.00	0.002*
#9 How to provide thermal care for a baby	2.37 ± 0.68	3.00 ± 0.00	0.002*
#10 How to advise about breastfeeding	2.74 ± 0.45	3.00 ± 0.00	0.025*
#11 How to assess when a baby is ready for discharge	2.63 ± 0.50	3.00 ± 0.00	0.008*
#12 How to determine when a baby needs antibiotics and transfer to a hospital	2.42 ± 0.51	3.00 ± 0.00	<0.001*
#13 How to give parents guidance for home care	2.47 ± 0.61	3.00 ± 0.00	0.003*

* denotes statistical significance at the 95% confidence level

**Figure 2:**
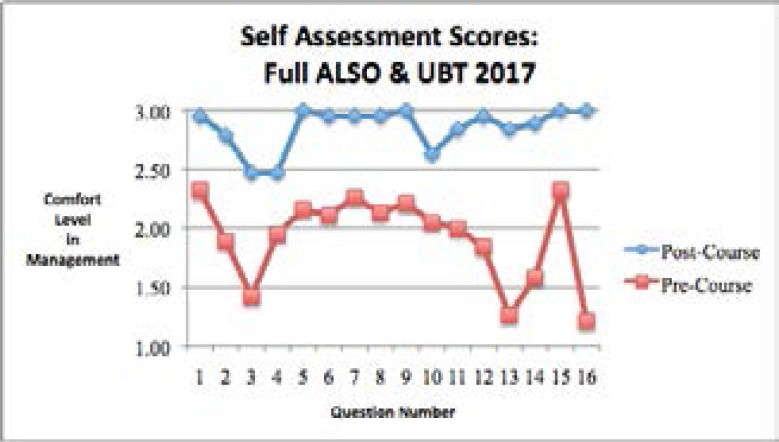
Self-assessment questions in the pre-training time period and the post-training time period for the full 2017 Advanced Life Support in Obstetrics (ALSO) and Uterine Balloon Tamponade (UBT) courses.

The 2017 self-assessment survey results from the full ALSO course and UBT training showed that the comfort level in diagnosing and managing obstetric emergencies was significantly higher in the post-course assessment compared to the pre-course assessment for all 16 self-assessment questions (Table S2 and Figure 2).

**Table S2:** Self-assessment questions in the pre-training time period and the post-training time period for the 2017 full Advanced Life Support in Obstetrics (ALSO) and Uterine Balloon Tamponade (UBT) classes

Self-Assessment	Pre-training assessment (n=19) Mean ± SD	Post-training assessment (n=19) Mean ± SD	p-value
#1 Miscarriage	2.32 ± 0.58	2.95 ± 0.23	0.001*
#2 Ectopic Pregnancy	1.89 ± 0.57	2.79 ± 0.42	<0.001*
#3 Gestational trophoblastic disease	1.42 ± 0.51	2.47 ± 0.51	<0.001*
#4 Gestational hypertension	1.95 ± 0.62	2.47 ± 0.51	0.027*
#5 Preeclampsia	2.16 ± 0.50	3.00 ± 0.00	<0.001*
#6 Eclampsia	2.11 ± 0.57	2.95 ± 0.23	<0.001*
#7 Vaginal bleeding in the third trimester	2.26 ± 0.56	2.95 ± 0.23	<0.001*
#8 Preterm Labor	2.13 ± 0.66	2.95 ± 0.23	<0.001
#9 Premature rupture of membranes	2.21 ± 0.63	3.00 ± 0.00	<0.001
#10 Interpreting fetal heart rate tracings	2.05 ± 0.62	2.63 ± 0.68	0.002*
#11 Labor dystocia (failure to dilate or descend in labor)	2.00 ± 0.67	2.84 ± 0.37	0.001*
#12 Breech presentation	1.84 ± 0.60	2.95 ± 0.23	<0.001*
#13 Vacuum or forceps assisted delivery	1.26 ± 0.45	2.84 ± 0.37	<0.001*
#14 Shoulder dystocia	1.58 ± 0.51	2.89 ± 0.32	<0.001*
#15 Postpartum hemorrhage	2.32 ± 0.58	3.00 ± 0.00	<0.001*
#16 Uterine Balloon Tamponade	1.21 ± 0.42	3.00 ± 0.00	<0.001*

* denotes statistical significance at the 95% confidence level

**Figure 3:**
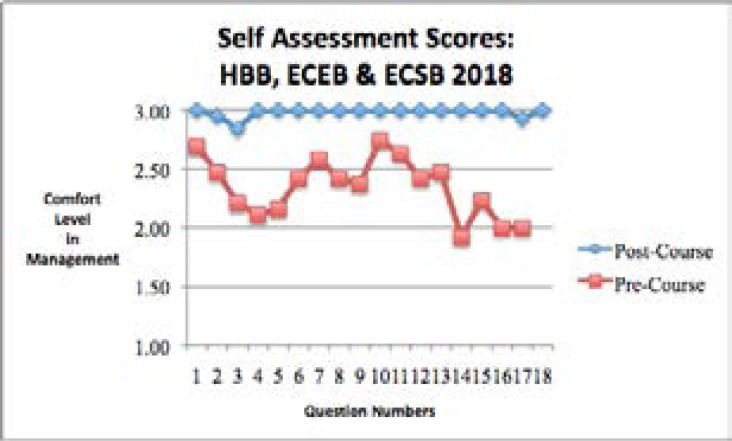
Self-assessment questions in the pre-training time period and the post-training time period for the 2018 Helping Babies Breathe (HBB), Essential Care for Every Baby (ECEB) and Essential Care for Small Babies (ECSB) classes.

The 2018 self assessment survey results from the HBB, ECEB and ECSB courses showed that the comfort level in performing important delivery actions was significantly higher in the post-course assessment compared to the pre-course assessment for all 18 self-assessment questions (Table S3 and Figure 3).

**Table S3:** Self-assessment questions in the pre-training time period and the post-training time period for the 2018 Helping Babies Breathe (HBB), Essential Care for Every Baby (ECEB) and Essential Care for Small Babies (ECSB) classes

Self-Assessment	Pre-training assessment (n=14) Mean ± SD	Post-training assessment (n=14) Mean ± SD	p-value
#1 Drying and stimulating a baby at birth	2.64 ± 0.50	3.00 ± 0.00	0.025*
#2 Using a checklist to prepare for birth	2.29 ± 0.73	2.93 ± 0.27	0.009*
#3 How to clean the bag and mask and suction after use	2.21 ± 0.70	2.93 ± 0.27	0.005*
#4 Management of baby that is not crying after birth	2.00 ± 0.71	3.00 ± 0.00	0.002*
#5 How to ventilate a baby with a bag and mask	2.21 ± 0.43	3.00 ± 0.00	0.001*
#6 How to provide eye care for a baby after birth	2.50 ± 0.65	3.00 ± 0.00	0.015*
#7 How to provide cord care for a baby after birth	2.33 ± 0.49	3.00 ± 0.00	0.005*
#8 How to protect babies from serious bleeding after birth	2.25 ± 0.75	3.00 ± 0.00	0.009*
#9 How to provide thermal care for a baby	2.71 ± 0.47	3.00 ± 0.00	0.046*
#10 How to advise about breastfeeding	2.54 ± 0.52	2.93 ± 0.27	0.014*
#11 How to assess when a baby is ready for discharge	2.15 ± 0.80	2.93 ± 0.27	0.009*
#12 How to determine when a baby needs antibiotics and transfer to a hospital	2.14 ± 0.77	3.00 ± 0.00	0.003*
#13 How to give parents guidance for home care	2.36 ± 0.00	2.93 ± 0.27	0.005*
#14 How to classify a small / premature baby as well or unwell based on breathing, temperature, weight, and feeding	1.92 ± 0.64	3.00 ± 0.00	0.002*
#15 How to keep a small / premature baby from getting cold.	2.23 ± 0.83	3.00 ± 0.00	0.009*
#16 Proper breastfeeding positioning and attachment for a small / premature baby	2.00 ± 0.55	3.00 ± 0.00	<0.001*
#17 Expressing breast milk and feeding a small / premature baby with a cup.	2.00 ± 0.68	2.93 ± 0.27	0.002*
#18 Correct nasogastric placement and nasogastric tube feeding	1.29 ± 0.47	3.00 ± 0.00	<0.001*

* denotes statistical significance at the 95% confidence level

**Figure 4:**
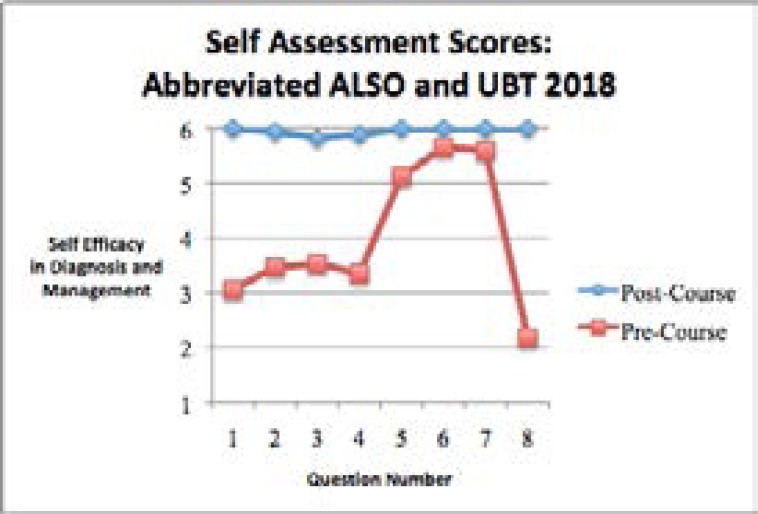
Self-assessment questions in the pre-training time period and the post-training time period for the abbreviated 2018 Advanced Life Support in Obstetrics (ALSO) and Uterine Balloon Tamponade (UBT) courses.

The 2018 self-assessment survey results from the abbreviated ALSO course and UBT training showed that the self efficacy in diagnosing and managing obstetric emergencies was significantly higher in the post-course assessment compared to the pre-course assessment for all 8 self-assessment questions (Table S4 and Figure 4).

**Table S4:** Self-assessment questions in the pre-training time period and the post-training time period for the 2018 abbreviated ALSO and UBT courses

Self-Assessment	Pre-training assessment (n=17) Mean ± SD	Post-training assessment (n=17) Mean ± SD	p-value
#1 I can describe the risk factors of and signs of shoulder dystocia	3.06 ± 1.75	6.00 ± 0.00	<0.001*
#2 I can list 3 ways to assist delivery when there is a shoulder dystocia	3.47 ± 1.46	5.94 ± 0.24	<0.001*
#3 I can describe at least 3 reasons it would NOT be safe to attempt a breech delivery	3.53 ± 1.42	5.82 ± 0.53	<0.001*
#4 I can describe the recommended steps in delivering a breech baby	3.35 ± 1.62	5.88 ± 0.33	<0.001*
#5 I know 3 methods to help prevent postpartum hemorrhage (active management of the 3^rd^ stage of labor)	5.12 ± 1.11	6.00 ± 0.00	0.003*
#6 I can list 4 causes of postpartum hemorrhage	5.65 ± 0.61	6.00 ± 0.00	0.025*
#7 I know the names and doses of at least 2 medications to give to control postpartum hemorrhage	5.59 ± 0.80	6.00 ± 0.00	0.046*
#8 I know how to assemble and place a uterine balloon tamponade for postpartum hemorrhage	2.18 ± 1.74	6.00 ± 0.00	<0.001*

* denotes statistical significance at the 95% confidence level

The evaluations completed by the learners at the end of the course demonstrated an overall very positive reception of the trainings. One midwife stated, “*I can now resuscitate well a distressed baby. Care for small babies and have knowledge and well contented with newborn care and handling obstetric emergencies and referring in case of advanced care*.” Another wrote, “*The Helping Baby Survive courses helped me to identify most of the gaps that I had and at least there is a greater change skills and knowledge have been acquired and I will make sure this knowledge will be passed to my fellow staff to save the community.*”

## Discussion

The post-course surveys showed significantly higher self efficacy in diagnosis and management as compared to the pre-course surveys in all questions in both the 2017 and the 2018 courses. This is consistent with multiple articles in the literature documenting the benefits of the HBB, ECEB, ECSB, ALSO and UBT courses.

If Uganda is going to meet the 2020 HDSP and 2030 SDG goals for maternal and neonatal mortality rates, significant improvements need to be made in closing the knowledge and skill gaps of birth attendants. Both the ALSO and HBS curriculum offer evidence based curriculum that incorporates adult learning theory and it would ideal to have all Ugandan providers train in both courses. However, given the shortage of midwife providers in the country, especially in rural areas, allowing providers enough time away from clinic to attend the full training for both courses is a practical challenge. Furthermore, while the ultimate goal is to create a self-sustaining robust instructor training programs in Uganda to train local providers to lead training courses, there is a still a current need for foreign providers as instructors. While many foreign providers welcome the opportunity to do global service education work, they also have limited time they can be away from home country practices.

We demonstrated a model for teaching a combined curriculum that incorporates the highest yield components in a practical, achievable, and efficient manner. In 2018, by cutting down on the ALSO course lectures, which was more passive learning than the simulation training, we were able to cover the entire HBB, ECEB, ECSB, key ALSO hands-on workshops and UBT course within a 5 day workweek. We would like to emphasize that the predominantly hands on design of the course minimized the potential for any verbal communication barriers. We were also still able to include practical examinations to assess learning at the end of each course in the allotted time. We learned through feedback we received that if the course had been any longer, we would have had to incorporate an additional day off for learners to study / relax. All of the learners' pre and post surveys showed a statistically significant increase in self-assessment of confidence and knowledge. Furthermore, the evaluation comments were overwhelmingly positive, highlighting that the learners found the courses to be both useful and applicable to their clinical practice.

Given the goal of improving efficiency, it was determined to do all hands-on sessions, but eliminate the non-hands-on ALSO sessions. Further, it should be noted that small groups training sessions are more effective than large scale training sessions. Therefore, this study can be considered a feasibility study and be a basis for a larger implementation project.

It should be noted that the participants needed to leave their duty stations to receive the training sessions. Future training models that can be considered may include low-dose, high-frequency models.^11^,^12^ It is well known that knowledge decays without refresher training. It is the authors' belief that a condensed annual refresher training can be accomplished. We suggest that future studies can develop 1-day or 2-day training regimens to maximize both training efficiency and midwife capability.

Limitations in this study exist in that efficacy of the course was assessed by learners' subjective self-assessment scores, subject to acquiescence bias in attempt to please the instructors, rather than objective assessment of application of the course material to clinical practice and improvement in maternal and neonatal outcomes. Specifically, the measures of learner confidence and their self-assessments of knowledge are insufficient to demonstrate effectiveness of an educational intervention There is current ongoing data collection being conducted by ECUREI to assess the latter and will be presented in future publications. Future studies can evaluate this implementation more formally with a complete framework.

## Conclusion

Like many developing countries, Uganda faces challenges of high maternal and neonatal mortality rates. Skilled birth attendants with the appropriate peripartum education may help to overcome this challenge. In this study, we found that the HBB, ECEB, ECSB, abbreviated (workshops only) ALSO and UBT could all be taught in a 5 day session with significantly improved self efficacy in diagnosing and managing a wide range of peripartum conditions.
